# Thidiazuron decreases epithelial‐mesenchymal transition activity through the NF‐kB and PI3K/AKT signalling pathways in breast cancer

**DOI:** 10.1111/jcmm.16079

**Published:** 2020-11-07

**Authors:** Peramaiyan Rajendran, Rebai Ben Ammar, Fatma J. Al‐Saeedi, Maged Elsayed Mohamed, MIH Islam, Saeed Y. Al‐Ramadan

**Affiliations:** ^1^ Department of Biological Sciences College of Science King Faisal University Al‐Ahsa Saudi Arabia; ^2^ Laboratory of Aromatic and Medicinal Plants Center of Biotechnology Technopole of Borj‐Cedria Hammam‐Lif Tunisia; ^3^ Department of Nuclear Medicine Faculty of Medicine Kuwait University Safat Kuwait; ^4^ Department of Pharmaceutical Sciences College of Clinical Pharmacy King Faisal University Al‐Ahsaa Saudi Arabia; ^5^ Department of Pharmacognosy Faculty of Pharmacy University of Zagazig Zagazig Egypt; ^6^ Department of Anatomy College of Veterinary Medicine King Faisal University Al‐Ahsa Saudi Arabia

**Keywords:** breast cancer, epithelial‐mesenchymal transition, matrix metalloproteinase, metastasis, Thidiazuron

## Abstract

Breast cancer is the major type among the women population globally. The treatment of cancer metastasis has made modest progress due to multiple factors. Thidiazuron (TDZ) is a novel plant growth regulator that has been shown to have anticancer effects. Therefore, we explored the anti‐metastatic potentials of TDZ in cell lines by assessing its potential to suppress the epithelial‐mesenchymal transition (EMT). We pretreated the BEAS‐2B and breast cancer (MDA‐MB‐231) cells with TDZ and deliberated alteration in a cell viability, mammosphere, migration, NF‐кB signalling, PI3K/AKT signalling and matrix metalloproteinase (MMP) expression and analysed the EMT induction by TGF‐β/TNF‐α‐stimulated BEAS‐2B cells. Treatment with TDZ (5‐50 μmol) diminished the migration and invasion of the extremely metastatic MDA‐MB‐231 cells. Additionally, TDZ treatment led to down‐regulation of uPAR, uPA, VEGF and MMP‐2/‐9 expression and up‐regulation of TIMP‐1/2 expression in these cells. Furthermore, TDZ treatment blocked invasion and EMT in non‐tumorigenic BEAS‐2B epithelial cells stimulated with TGF‐β/TNF‐α.TDZ prevents EMT and may thus block metastasis of breast cancer cells.

## 1 INTRODUCTION

Breast cancer is a recurrently identified cancer type in the world, causing women 523 000 deaths and 15.1 million illness‐attuned lifetimes in 2015.^1^ Advances made in the field of breast cancer over the last few years have augmented the 5‐year survival rate of victims worldwide.^2^ Despite this significant progress, the efficient treatment approaches for metastatic breast cancer remain as an imperative cause of deaths worldwide.^3^ It might seem the elevation of the expectation to treat the metastatic breast cancer, an ailment that is obstinately resistant to finest efforts of modern drug. The epithelial‐mesenchymal transition (EMT) was identified as a decisive process for carcinoma development and metastases.^4^

Epithelial‐mesenchymal transition is a mechanism in that the polarized epithelial cells in tumours drop their polarity and ability to form cell adhesions and become mesenchymal stem cells, which are characterized by increased migratory ability, invasiveness, metastatic potential and drug resistance.^4^, ^5^ The Snail and Slug signalling cascades are in the EMT of cancer cells.^6^, ^7^ In various cancers, Snail expression is associated with AKT/GSK or NF‐кB activity and promotes cell invasion and migration.^8^ Specifically, Snail and Slug are transcription factors, which mediate the EMT via binding to the E‐boxes in EMT target genes like the classical epithelial cell adhesion molecule E‐cadherin, entirely absent in mesenchymal cells.^9^

Besides decreasing the E‐cadherin expression, the binding of Slug and Snail to E‐boxes in a E‐cadherin augments the matrix metalloproteinase‐9 (MMP‐9) activity, a protease that promotes cell invasion.^10^, ^11^ MMP‐9 performs a vital function in metastasis via destroying extracellular matrix (ECM) and thus enabling the migration as well as invasion of cancer cells. EGF induces the expression in cancer cells via stimulating the signalling pathways PI3K/AKT and NF‐kB.^12^, ^13^, ^14^ Numerous preceding researches have explored the effects of inhibiting PI3K on cancer metastasis.^12^, ^15^ LY294002 and wortmannin are strong PI3K inhibitors, which are normally utilized in laboratory and preclinical researches but have been prohibited from clinical purpose utilization because of their unsteadiness, reduced solubility and/or augmented toxicity. However, few studies on cancer treatments have focused on the suppression of suppression of EMT‐associated pathways and/or the mechanisms responsible for EMT inhibition.

Bioactive substances display a number of anticancer features, like repression of cell multiplication, activation of apoptosis and cancer metastasis. Several studies have demonstrated their significant potential to treat the lung cancer or triple‐negative breast cancer (TNBC) via inhibiting the tumour and metastasis.^16^, ^17^ The less toxicity of these compounds makes them as promising agents for the treatment of cancer.

Thidiazuron (TDZ) is a phytochemical with cytokinin‐like activity that has been extensively used as an herbicide and a plant growth regulator.^18^, ^19^, ^20^ In a study of the cytotoxic effects of TDZ in HeLa cervical cancer cells, Enkhtaivan et al mentioned that the toxicity of TDZ in cancer cells was increased when evaluated with normal cells.^21^ This effect was accompanied via the presence of DNA breaks, loss of mitochondrial membrane potential, and altered several apoptosis‐related gene expression.^21^ Importantly, TDZ was found to target caspase‐3 in molecular docking simulation studies.^21^ Based on the above study, we need to inspect whether TDZ could inhibit EMT and metastases in MDA‐MB‐231 cells.

In this investigation, we aimed to ascertain the potential of TDZ to inhibit metastasis, EMT and associated changes in breast cancer. In that order, we used a validated EMT and metastasis MDA‐MB‐231 cell‐based model. EMT and the chief molecular markers concerned were inspected to characterize the anti‐EMT and anti‐metastatic properties of TDZ.

## 2 MATERIALS AND METHODS

### 2.1 Reagents and antibodies

Thidiazuron and 3‐(4,5‐dimethylthiazol‐2‐yl)‐2,5‐diphenyltetrazolium bromide reagent for the MTT assay were attained from Sigma‐Aldrich (St. Louis, MO, USA). The penicillin‐streptomycin‐neomycin, and Dulbecco's‐modified Eagle's F‐12 medium (DMEM/F12) were acquired from GIBCO BRL/Invitrogen. Antibodies specific for MMP9, MMP2, uPAR, uPA, TIMP1, TIMP2, caspase‐3, PAI‐1, NF‐κB (p65), phos‐IKK, VEGF, PARP, p‐AKT, AKT, p‐PI3K, PI3K, lamin‐B1, β‐actin and IKK were bought from Thermo Scientific. F‐actin (Alexa Fluor 488 Phalloidin) was purchased from Thermo Scientific.

### 2.2 Cell culture

MDA‐MB‐231, HepG2 and MCF‐7 cells were bought from Cell Line Service (CLS, Germany), and the A549 cell line and the normal BEAS‐2B cells were acquired from American Type Culture Collection (ATCC; USA). A549, MCF‐7 and MDA‐MB‐231 cells were seeded in RPMI medium loaded along with heat‐inactivated FBS (10%) penicillin‐streptomycin‐neomycin (1%), and l‐glutamine (2 mmol/L) and maintained beneath the typical situations (5% CO2, 37°C, 95% moisture), while HepG2 and BEAS‐2B cells were seeded in DMEM/F12 consists of heat‐inactivated FBS (10%), glutamine (2 mmol/L), hydrocortisone (0.5 μg/mL), penicillin‐streptomycin‐neomycin (1%), insulin (10 μg/mL) and human EGF (20 ng/mL) under the same incubator conditions. After the cultures were harvested, the cells were counted with the aid of the haemocytometer, and morphological alterations were inspected via the phase‐contrast microscope at the 200× magnification.

### 2.3 MTT assay

A MTT assay was used to test the viability of MDA‐MB 231, MCF‐7, A549, HepG2 and BEAS‐B cells to detect NADH‐dehydrogenase activity as elaborated previously {Hseu, 2019 #86}.

### 2.4 In vitro wound‐healing activity by Scratch assay

The wound‐healing assay was administered to test cell migration. MDA‐MB231 cell monolayers were scratched and treated with CLG according to the previously described protocol {Hseu, 2019 #87}.

### 2.5 Cell invasion assay

The invasion assay was carried out using BD Matrigel invasion chambers (Bedford, MA, USA) as stated earlier in the protocol {Hseu, 2019 #87}.

### 2.6 RNA extraction and Real Time‐PCR analysis

Using TRIzol, complete RNA was extracted and PCR was performed as previously established{Kim, 2014 #89}. The sequences of primer utilized in this investigation were as follows: E‐cadherin forward, 5′‐TGGGTTATTCCTCCCATCAG‐3′, reverse, 5′‐TTTGTCAGGGAGCTCAGGAT‐3′ (Sehrawat and Singh, 2011); Snail: forward, 5′‐CGAAAGGCCTTCAACTGCAAAT‐3′, reverse,5′‐ACTGGTACTTCTTGACATCTG‐3′ (Hseu YC); and GAPDH forward, 5′‐GAACGGGAAGCTCACTGGCATGG‐3′, reverse, GCCCTCCGACGCCTGGTTCAC.

### 2.7 Mammosphere formation assay

The mammospheres were seeded in a low adherence with CnT‐27, serum‐free medium and growth factors (CellnTEC Advanced Cell Systems, Bern, Switzerland) as mentioned by Dontu et al. The self‐renewal capacity of MDA‐MB‐231 cells was inspected via generating additional mammospheres. At the every process, spheres were estranged into single cells and re‐plated beneath the previously used low‐adherence situations. Shortly, mammospheres were gathered and centrifuged. Later than the supernatant aspiration, 0.1% trypsin/EDTA was amalgamated to cell pellet, then pellet was resuspended, and the mixture was maintained for 1 minute at 37°C. The suspension was then sifted via a 70‐µm sifter, and the cells were counted and re‐plated in the medium and then mammospheres were being inspected through the microscopy.

### 2.8 Western blotting study

Cells were loaded at 1 × 10^6^ cells/6‐cm plate and subsequently pretreated with TDZ at concentrations ranging from 5 to 50 μmol for 24 hours. After the administration, the cells were scraped and cleansed in a chilled PBS; afterwards, the nuclear, cytoplasmic and total extracts were prepared as mentioned earlier.^56^ For the specified concentrations and time‐points, the cells were treated with TDZ and Western blotting was performed as stated earlier {Lee, 2020 #88}.

### 2.9 Immunofluorescence staining

Cells were grown in 8‐well plate (1 × 10^4 ^cells per well density), and the cells were subjected to TDZ (50 μmol) pre‐supplementation for the 24 hours. Then, cells were processed for 15 minutes with 2% of paraformaldehyde, permeabilized via Triton X‐100 (0.1%) for 10 minutes, cleansed and blocked through 10% of FBS/PBS. After that, they were maintained with anti‐p65 primary antibodies in a 1.5% FBS solution. Then, cells were sustained with FITC‐conjugated secondary antibodies diluted in 6% BSA/PBS for 1 hour. Afterwards, the cells were stained with 1 μg/mL DAPI for 5 mins. Later than two cleanses with PBS for 5‐10 minutes each, the cell arrangements were cleansed with distilled water, fixed in ethanol for 5 minutes and fixed with Fluoromount G (Southern Biotech; attained via Biozol Diagnostica, Eching, Germany). Lastly, immunofluorescence microscopy images were obtained with a Leica D6000 fluorescence microscope (Leica, Germany) outfitted with an AxioCam HR (Carl Zeiss).

### 2.10 Gelatin zymography assay

The activities of MMP‐2 and −9 in the MDA‐MB‐231 cells were inspected through the gelatin zymography protease technique. Briefly, 3 × 10^5^ MDA‐MB‐231 cells was loaded to 12‐well plates consisting of DMEM supplemented with FBS (10%) and grown to a near‐confluency. Subsequently, cells were trypsinized and reseeded in DMEM and incubated with TDZ (25‐50 μmol) for 24 hours. Then, a right amount of the gathered medium was assorted with SDS sample buffer but not subjected to either a boiling or reducing step. For the analysis of MMPs, the SDS‐PAGE (8%) gel was supplemented with 1 mg/mL gelatin (casein), and the sample was resolved by electrophoresis.

Zymography protease assays were also utilized to inspect the MMP‐2 and MMP‐9 activities in a TGF‐β/TNF‐α‐induced BEAS‐2B cells. Briefly, BEAS‐2B cells (1 × 10^6^ cells/well) were loaded to 6‐well plates in a FBS (10%) supplemented medium and grown to a monolayer confluence. Cells were reseeded in a medium and incubated along with TGF‐β/TNF‐α (10 ng/mL) and TDZ (25 μmol) for 24 hours. All subsequent procedures were performed as described above. Alterations in the MMP‐2 and ‐9 activities were calculated by MatrixInspector 2.1 tool (AlphaEase, Genetic Technology, Inc).

### 2.11 Actin distribution and cell morphology analysis

Unstimulated BEAS‐2B cells at 1 × 10^4^ cells/well population were seeded in DMEM medium that restrained FBS (10%). Later than 24 hours, the cells were pre‐supplemented with TDZ (25 μmol) for 1 hour and subsequently triggered with TGF‐β/TNF‐α (10 ng/mL) for 24 hours. Then, the cells were mounted on 3.7% of paraformaldehyde, blocked in 3% of BSA and stained for visualization of F‐actin by TRITC‐conjugated phalloidin. Then, cells were stained with DAPI (1 μg/mL), as described in section 2.9. Lastly, immunofluorescence microscopy pictures were monitored with a Leica D6000 fluorescence microscope (Leica, Germany) outfitted with an AxioCam HR (Carl Zeiss).

### 2.12 DNA fragmentation assay

To evaluate the DNA fragmentation in MDA‐MB −231 cells upon TDZ treatment was carried out through Cell Death Detection ELISA PLUS kit (Roche Applied Science) by the procedures of the manufacturer and as mentioned earlier.^22^

### 2.13 Statistical analyses

Experimental data were articulated as a mean ± SD. ANOVA was utilized to analyse every investigational result, and Dunnett's test was used for pairwise comparisons. Variations among the test groups were regarded as significant at *P* < .05.

## 3 RESULTS

### 3.1 TDZ suppresses the viability of cell lines

**Figure 1:**
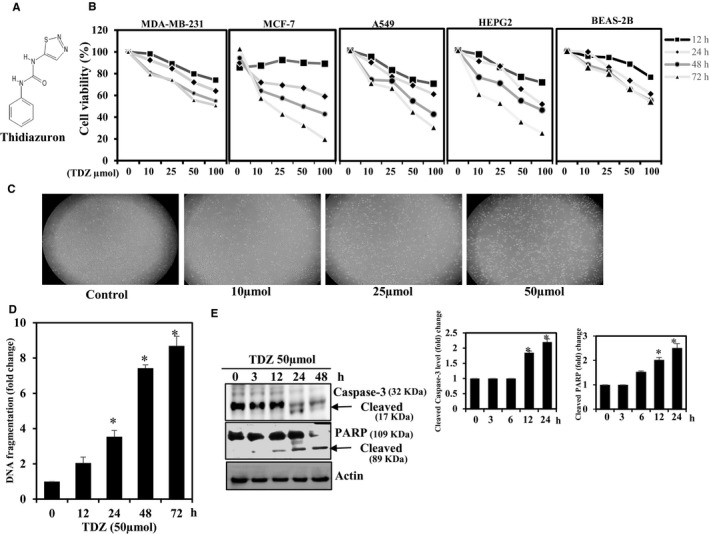
Thidiazuron (TDZ) inhibit cell growth and induced apoptosis in MDA‐MB‐231 cancer cells. A, The structure of TDZ is shown. B, MDA‐MB‐231, MCF‐7, A549, HepG2 and normal lung epithelial BEAS‐2B cells were supplemented with TDZ (0‐100 μmol) or a vehicle control (0.1% DMSO) for different time periods, and cell viability was assessed with MTT assays. C, MDA‐MB‐231 cells were supplemented with TDZ (0‐50 μmol) for 24 h, and cell morphology was examined beneath the phase‐contrast microscope. D, MDA‐MB‐231 cells were supplemented with 50 μmol TDZ. DNA fragmentation was analysed by measuring photometric at various time‐points (0‐72 h). E, MDA‐MB‐231 Cells were supplemented with TDZ (50 μmol) and harvested at different time‐points (0‐48 h). Then, the levels of apoptosis protein markers were analysed by Western blotting. β‐actin was utilized as the internal control. The data are articulated as the mean ± SD of triplicate measurements, *P < .05 when evaluated with control cells

We began our assessment of the anticancer effects of TDZ (Figure 1A) by assessing the effect of TDZ treatment on cancer cells evaluated to that of normal cells. Thidiazuron (Figure 1B) decreased the viability of metastatic and non‐metastatic cancer cells (MDA‐MB‐231, MCF‐7, A549 and HepG2) and epithelial cell line (BEAS‐2B) in a time‐ and dose‐reliant mode (Figure 1B). These outcomes indicated that TDZ was more potent against MDA‐MB‐231 cells than against epithelial cells. Also, the supplementation of MDA‐MB‐231 cells with an diverse dosages of TDZ (5‐50 μmol) for 24 hours resulted in decreased cell proliferation and cells were less elongated (Figure 1C), suggesting that TDZ has powerful anticancer effect.

### 3.2 TDZ promotes DNA fragmentation and caspase‐3 and PARP cleavage in MDA‐MB‐231 cells

Apoptosis is a critical mechanism, which helps to reduce cell growth.^22^ The augmented DNA fragmentation was noticed in a TDZ (50µmol)‐treated MDA‐MB‐231 cells as determined (Figure 1D). Additionally, TDZ treatment resulted in a time‐reliant stimulation of caspase‐3, manifested as cleaved caspase‐3, and cleavage of PARP protein (Figure 1E). The cleavage of caspase‐3 and PARP were statistically significant after 12 and 24 hours of incubation with TDZ. These outcomes reveal that TDZ can stimulate the apoptosis of MDA‐MB‐231 cells.

### 3.3 TDZ inhibits MDA‐MB‐231 cell migration and invasion

**Figure 2:**
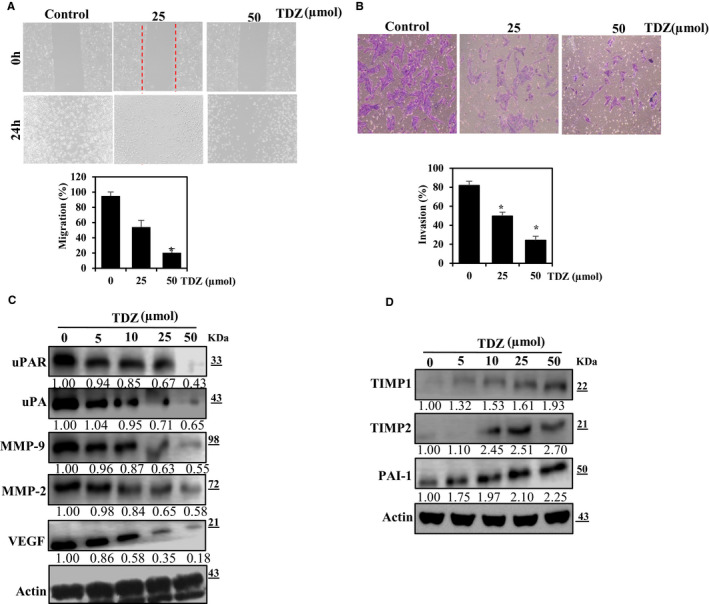
TDZ inhibits human breast cancer cell proliferation, cell migration and invasion. A, MDA‐MB‐231 cell monolayers were scratched, and cells were supplemented with TDZ (25 and 50 µmol) for 24 h. Migration was noticed with an optical microscope (200× magnification) by a wound‐healing assay. Commercially available MRI wound‐healing tool was used to calculate the area of the wound and assess wound closure. B, MDA‐MB‐231 cells supplemented with TDZ (0‐50 µmol) were loaded to the upper chambers of Matrigel‐coated transwells, and invasion was inspected via total cell counting, which had crossed to the lower chamber after 24 h The inhibition percentage of invasion was quantified and is expressed relative to the control (untreated cells), whose level of invasion was set at 100%. Invading cells quantified by using manual counting. C, D, Alterations in the status of metastasis‐associated proteins in respond to the TDZ supplementation were inspected via Western blot. MDA‐MB‐231 cells were supplemented with TDZ (0, 5, 10, 25, or 50 μmol) for 24 h. Β‐actin was utilized as controls. C, Effects of TDZ supplementation on the status of MMP‐2, MMP‐9, uPA, uPAR and VEGF. D, Effects of TDZ supplementation on the statuses of the endogenous inhibitors of the proteins shown in (C), namely TIMP‐1, TIMP‐2 and PAI‐1. The data are articulated as the mean ± SD of triplicate values. *P < .05, when evaluated with control

Wound healing is a critical mechanism in that the skin renews itself followed by the damages, and this process was mentioned to be associated to EMT and tumorigenesis.^23^ We inspected the inhibitory potential of TDZ on cell migration by performing a wound‐healing analysis. Untreated MDA‐MB‐231 cells revealed appreciable migratory potential, while wound closure by cells treated with 25 or 50 μmol TDZ was delayed (Figure 2A). Furthermore, the gap difference was significantly reduced to exposure to 25 or 50 μmol TDZ, whereas wound closure in the control group (Figure 2A). Next, we measured the ability of TDZ to inhibit MDA‐MB‐231 cell invasion by performing Matrigel invasion assays. The results showed that at doses of 25 and 50 μmol, TDZ appreciably inhibited cell invasion (Figure 2B). These findings indicate that TDZ was possessed the anti‐migratory and anti‐invasive potentials against the MDA‐MB‐231 cells.

### 3.4 TDZ down‐regulates the expression of uPA, uPAR, MMPs and VEGF

uPA and MMPs perform a crucial function in the degradation of basement membrane and are important mediators of migration and invasion.^24^ To determine whether the TDZ‐mediated effects on cell invasion and migration are linked with changes in the levels of uPA, uPAR, MMPs and VEGF, the levels of these proteins were inspected in MDA‐MB‐231 cells supplemented with TDZ (0‐50 μmol) for 24 hours. The results showed substantial dose‐dependent reductions in the uPA, uPAR, MMP‐2, MMP‐9 and VEGF expressions (Figure 2C). Thus, we measured the levels of TIMP1, TIMP2 and PAI‐1 in MDA‐MB‐231 cells that were supplemented with TDZ (5‐50 μmol) for 24 hours. The results showed substantial dose‐dependent increases in the status of TIMP‐1, TIMP‐2 and PAI‐1 (Figure 2D). Together, these findings indicate that TDZ may inhibit migration and invasion by modulating by down‐regulations relevant proteins.

### 3.5 TDZ attenuates NF‐κB activation by suppressing I‐κBα down‐regulation

**Figure 3:**
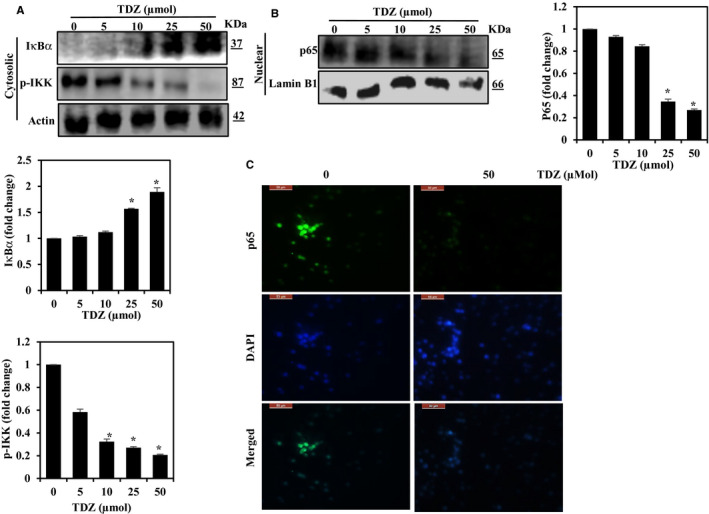
TDZ down‐regulation of NF‐kB signalling in MDA‐MB‐231 cells. A, Cytosolic fractionation and Western blot analysis of TDZ effects on I‐κB and p‐IKK levels. B, TDZ effects on p65 in nuclear extracts. MDA‐MB‐231 cells were supplemented with 0‐50 μmol TDZ for 2 h. Cytoplasmic and nuclear extracts were prepared by described as methods sections. Lamin B and β‐actin were utilized as the internal controls for the cytoplasmic and nuclear fractions. The data are articulated as the mean ± SD of triplicate values. *P < .05, when evaluated with control. C, Cells were grown on four‐chambered slides, exposed to TDZ (50 μmol) for 2 h, mounted and permeabilized. The cells were incubated with an anti‐p65 antibody subsequently, FITC‐labelled secondary antibody. The subcellular localization of p65 was examined through the confocal microscope at 40× magnification. The data are articulated as the mean ± SD of triplicate measurements. *P < .05 when evaluated with control

The NF‐κB signalling cascade is a critical regulator of the MMP, VEGF and uPA expression.^6^ So, we inspected the ability of TDZ on NF‐κB signalling in MDA‐MB‐231 cells. We noticed that reduced status of NF‐κB was appreciably reduced in cells treated with 25 and 50 µmol TDZ as indicated by decreased p65 levels in the nucleus, and the results for lamine B showed that were not affected (Figure 3B). Inhibition of nuclear translocation of p65 in response to TDZ treatment was confirmed by immunofluorescence analysis (Figure 3C). I‐κBα binds to NF‐κB preventing its transfer to the nucleus where it would exert its function. The degradation of I‐κBα releases p65 for translocation to the nucleus and therefore we measured the effect of TDZ treatment on I‐κBα cytoplasm levels.^12^ TDZ‐treated cells and found that statistically significantly reduced I‐κBα degradation (Figure 3A). Based on these results TDZ could degradation of the protein, which controls the stability of NF‐κB activation. We utilized Western blotting to investigate IKKα phosphorylation to decide if the I‐κBα degradation inhibition is an outcome of IKKα phosphorylation inhibition. Phosphorylated IKKα in the cytoplasmic fraction of cells supplemented with diverse dosages of TDZ and found that TDZ treatment appreciably reduced the levels of phosphorylated IKKα (Figure 3A). Overall these outcomes reveal that TDZ suppresses nuclear stimulation of NF‐κB probably by inhibiting I‐κBα degradation via a reduction in phosphorylated IKKα levels.

### 3.6 TDZ attenuates PI3K/AKT activation in MDA‐MB‐231 cells

**Figure 4:**
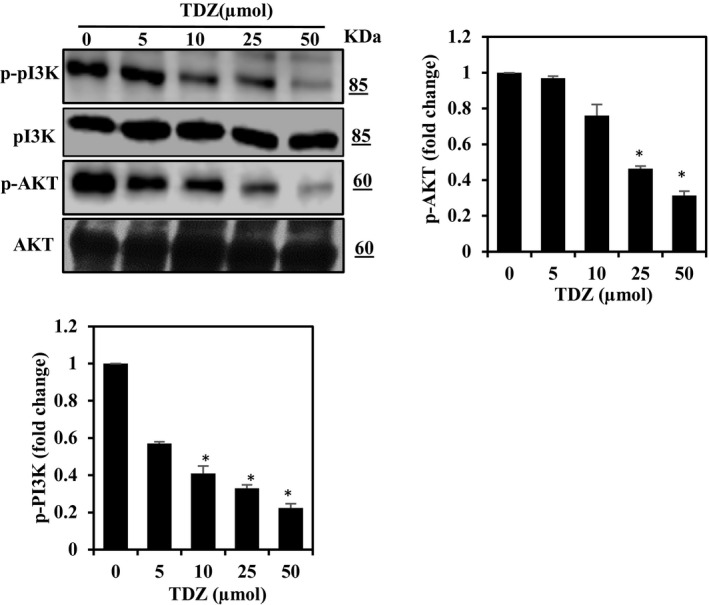
TDZ down‐regulation of PI3K/AKT signalling in MDA‐MB‐231. A, Phosphorylated PI3K (p‐PI3K) and AKT (p‐AKT) status was inspected via Western blotting. Cells were supplemented with TDZ *(*0‐50 μmol) for 24 h. β‐actin, total PI3K, and AKT statuses were used as internal controls. The data are articulated as the mean ± SD of triplicate measurements. *P < .05 when evaluated with control

Besides NF‐kB, MMP expression, and hence cancer cell migration, is also regulated by the PI3K/AKT family of transcription factors. Therefore, next we evaluated PI3K/AKT family of transcription factors performs a critical functions in encouraging cancer cell migration and stimulation of Akt, was linked with invasion and metastasis of tumours.^25^ So we further evaluated the effects of TDZ on PI3K and AKT phosphorylation by Western blot. Thidiazuron treatment significantly diminished p‐AKT and p‐PI3K levels and augmented PI3K levels in time‐reliant mode (Figure 4). The outcomes indicate that TDZ treatment suppresses the migratory and invasive potentials of MDA‐MB‐231 cells and that capacity was mostly regulated through the PI3K/AKT signalling cascade.

### 3.7 TDZ inhibits MMP‐9 via suppression of the PI3K/AKT and NF‐κB pathways in MDA‐MB‐231 cells

**Figure 5:**
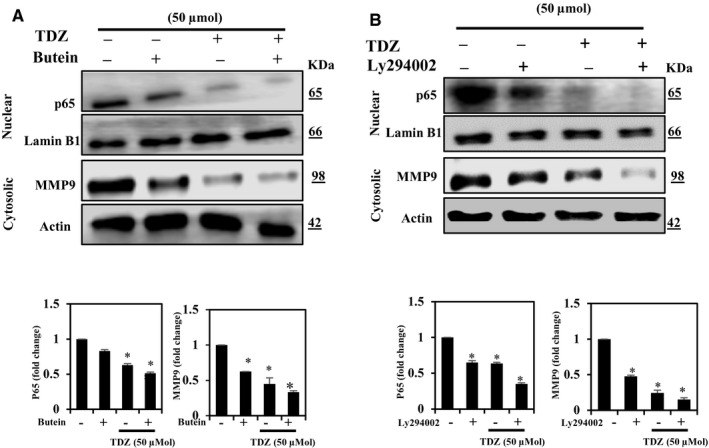
TDZ inhibits EMT through down‐regulation of MMP9 expression and NF‐κB signalling in MDA‐MB‐231 cells. A, TDZ effects on MMP‐9 expression and NF‐κB signalling are shown. Nuclear p65 levels (6 h) and MMP‐9 expression (24 h) were evaluated following treatment with TDZ (50 μmol) for 6 and 24 hours, respectively, in the absence or presence of the NF‐κB inhibitor Butein (50 μmol), and Western blot analysis was performed. β‐actin and Lamin B were utilized as internal controls. B, TDZ effects on NF‐κB/MMP‐9 expression after down‐regulation of PI3K/AKT signalling pathway activity. Nuclear p65 levels and MMP‐9 expression were evaluated following treatment with TDZ (50 μmol) for 6 and 24 h, respectively, in the absence or presence of the PI3K/AKT inhibitor LY294002 (30 μmol/L), and Western blot analysis was performed. β‐actin and Lamin B were utilized as controls. The data are articulated as the mean ± SD of triplicate measurements. *P < .05 when evaluated with control

NF‐κB performs a major function in the mediation of expression of MMP in cancer cells.^6^ The previous results showed that TDZ treatment down‐regulated MMP‐9 expression as well as NF‐κB and PI3K/AKT signalling. Since MMP‐9 expression is arbitrated through the NF‐κB that in turn is mediated through the PI3K/AKT, we next examined whether TDZ‐mediated down‐regulation occurred via NF‐κB inhibition and PI3K/AKT cascade. Consequently, first, we evaluated the potential of TDZ treatment on NF‐κB stimulation and MMP‐9 expression using MDA‐MB‐231 cells pretreated with butein. The outcomes were proved that TDZ decreased p65 and MMP‐9 protein levels and that these TDZ‐mediated effects were more pronounced with butein pretreatment (Figure 5A).

The PI3K/AKT signalling is a critical pathway concerned in NF‐κB/MMP‐9 stimulation. We have already shown that TDZ attenuates PI3K/AKT activation (see Figure 5B). To investigate the relationship between the PI3K/AKT pathway and TDZ‐mediated decreases in p65 and MMP‐9 levels, we assessed the impact of treatment with LY294002, MMP‐9 expression, on nuclear p65, a PI3K inhibitor, in a presence or absence of TDZ. We observed that in cells treated with LY294002 without TDZ, nuclear p65 levels and expression of MMP‐9 were lower than in those treated without LY294002 and TDZ (Figure 5B). Overall, these outcomes were revealed that TDZ suppresses MMP‐9 expression via PI3K/AKT and NF‐κB cascade inhibition in MDA‐MB‐231 cells.

### 3.8 TDZ inhibits the MMP expression stimulated by TNF‐α/TGF‐β activation of non‐tumorigenic cells

**Figure 6:**
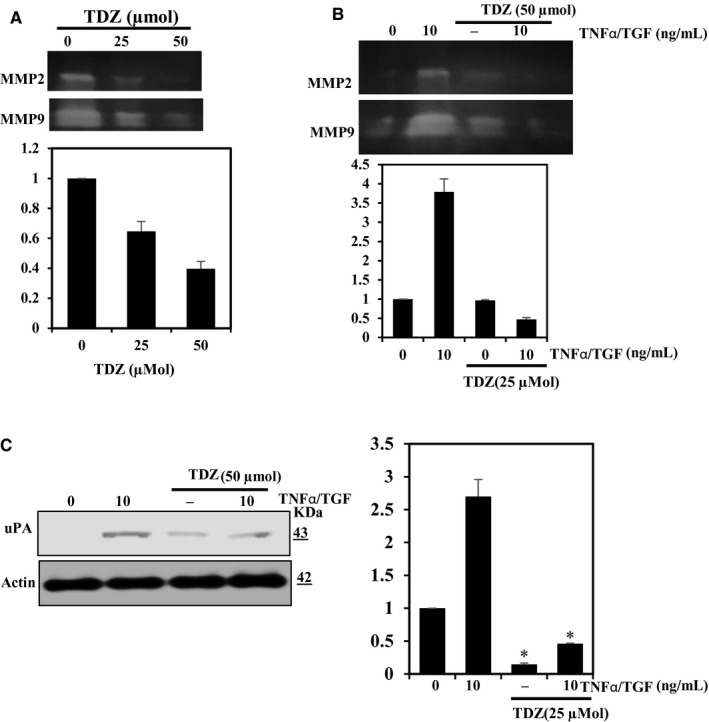
TDZ inhibits TGFβ/TNF‐α induced MMPs and uPA in BEAS‐2B cells and un‐ induced MBA‐MDA‐231. A, TDZ effects on MMP‐2 and MMP‐9 activity in a medium from MDA‐MB‐231 cells were inspected by gelatin zymography. B, TDZ effects on MMP‐2 and MMP‐9 activity in a medium from TNF‐α/TGF‐β‐stimulated BEAS‐2B cells were evaluated by gelatin zymography. C, TDZ effects on uPA activity in conditioned medium from TNF‐α/TGF‐β‐induced BEAS‐2B cells were evaluated by Western blotting. The data are articulated as the mean ± SD of triplicate measurements. *P < .05 when evaluated with control

Epithelial‐mesenchymal transition plays significant functions in a typical development, tissue fibrosis and metastasis. TGF‐α is the active EMT inducer and is suspected to lead to tissue fibrosis, which includes hepatic, renal and lung fibrosis. However, there is evidence that TNF‐α is related to the EMT.^26^ Therefore to inspect the potential of TDZ administration on proteins associated with EMT and metastasis, we treated TNF‐α/TGF‐β‐stimulated BEAS‐2B lung epithelial cells and unstimulated MDA‐MB‐231 cells with TDZ (50 μmol) and assessed the status of MMP‐2 and MMP‐9 function and uPA expression in these cells (Figure 6). The zymography analysis showed that TDZ pretreatment attenuated MMP‐2 and MMP‐9 actions (Figure 6A,B), and the Western blot investigation revealed that TDZ administration decreased uPA protein expression in TNF‐α/TGF‐β‐triggered BEAS‐2B cells (Figure 6C). Taken together, these outcomes were revealed that TDZ may inhibit TNF‐α/TGF‐β‐stimulated MMPs and uPA in BEAS‐2B cell lines.

### 3.9 TDZ suppresses EMT‐associated changes in gene expression by up‐regulating E‐cadherin in MDA‐MB‐231 cells

**Figure 7:**
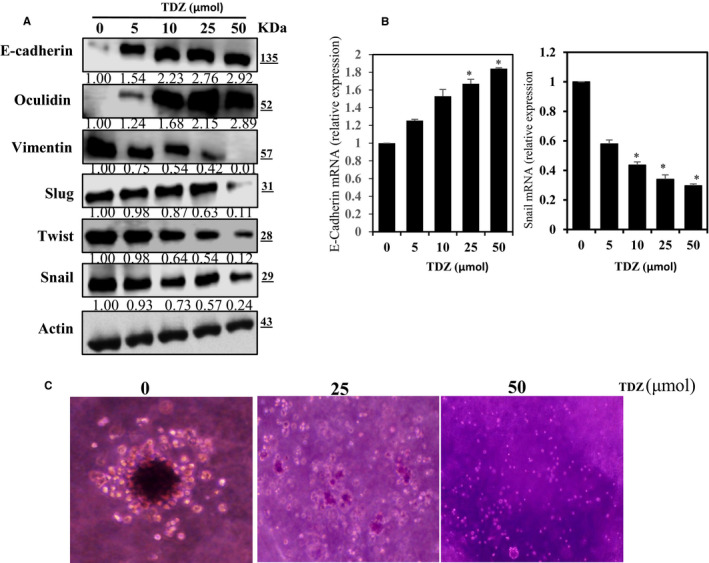
TDZ treatment decreases the expression of EMT‐related protein markers and increases E‐cadherin status in MDA‐MB‐231 cells. A, MDA‐MB‐231 cells were supplemented with TDZ (5‐50 μmol) for 24 h. TDZ effects on the protein status of epithelial markers E‐cadherin and Occludin and on mesenchymal markers Slug, Twist, Snail and Vimentin determined by Western blot test. B, RT‐PCR examination was performed to inspect the mRNA expression status of E‐cadherin and Snail after supplementation with TDZ (5‐50 μmol) for 24 h. GAPDH was utilized as the internal control. The data are articulated as the mean ± SD of triplicate measurements. *P < .05 when evaluated with control. C, TDZ effects on mammosphere formation are shown. MDA‐MB‐231 Cells were supplemented with TDZ (25 and 50 μmol) or vehicle control (0.1% DMSO) for 7 days. The mammosphere was imaged, counted and measured using an Motic AE2000 Inverted Phase Contrast Digital Tablet Microscope. The data are articulated as the mean ± SD of triplicate measurements. *P < .05 when evaluated with control

E‐cadherin is a key inhibitor of EMT. Thus, we sought to determine whether TDZ treatment suppresses EMT. We inspected the E‐cadherin expression in MDA‐MB‐231 cells. The cells were maintained with varying dosages of TDZ (5‐50 μmol) for 24 hours, and E‐cadherin levels were measured at the RNA and protein status. In TDZ‐treated MDA‐MB‐231 cells, we noticed a dose‐reliant augment in E‐cadherin and Occludin expression and a diminished Slug, Twist, Vimentin and Snail protein expressions (Figure 7A). Consistently, we observed a dose‐reliant augment in E‐cadherin mRNA expression (Figure 7B) and suppression in Snail expression in these cells (Figure 7B). Based on this result indicate that TDZ inhibit EMT via up‐regulation of E‐cadherin.

### 3.10 TDZ attenuates mammosphere formation

Mammospheres are spherical clusters of non‐adherent mammary stem cells. Mammosphere culture has been commonly used to supplement mammalian epithelial stem cells (CSCs) of the breast cancer. EMT also causes the characteristics of the stem cells in typical and transformed mammary cells. The mammary assay is ideal for revealing stem like characters in lines of MAD‐MB‐231 cells, which express E‐cadherin. A previous study showed that MAD‐MB‐231 cells express E‐cadherin.^27^ Thus, to determine whether TDZ affects mammosphere formation by MAD‐MB‐231 cells, we uncovered cells to numerous doses of TDZ for one week, and our findings indicated that TDZ reserved adherent spherical breast cancer cluster formation in vitro, as these cells become unable to generate secondary spheres (Figure 7C). These results indicate that TDZ is capable of a strongly inhibiting the formation of mammospheres by breast cancer cells.

### 3.11 TDZ inhibits the EMT stimulated by TNF‐α/TGF‐β

**Figure 8:**
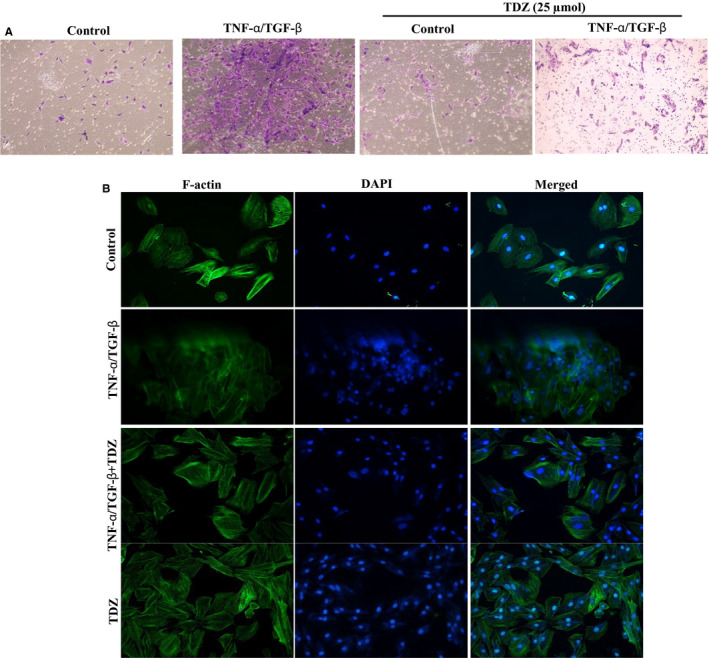
TDZ inhibits EMT‐related cell invasion and cytoskeletal rearrangement in BEAS‐2B cells. BEAS‐2B cells were pre‐supplemented with 25 μmol TDZ for 90 min and then activated with TGF‐β/TNF‐α (10 ng/mL) for 24 h. A, Cell invasion was measured via counting cells in three microscopic fields each sample. B, The cytoskeletal pattern of F‐actin was assessed through the immunofluorescence (100× magnification)

Lung is the frequent sites of distant breast cancer metastasis linked with deprived survival outcome for the victims. The abnormal expression of TGF‐β facilitates the development of breast cancer by altering the microenvironment. TGF‐β helps to establish a pre‐metastatic microenvironment for the lung by modulating some inflammatory cytokines and growth factors.^28^ Therefore, we required to inspect the anti‐EMT potential of TDZ (50 μmol/L) on TNF‐α/TGF‐β‐stimulated non‐tumorigenic BEAS‐2B. To this end, we performed invasion assays to measure BEAS‐2B cell invasion through a Matrigel layer. The outcomes demonstrated that TNF‐α/TGF‐β administration noticeably augmented the invasion of BEAS‐2B cells evaluated with normal cells. As well, TDZ pretreatment revealed substantial suppression of the TNF‐α/TGF‐β‐stimulated invasion of BEAS‐2B cells (Figure 8A). Further, we examined the F‐actin distribution. Cells administered with TNF‐α/TGF‐β appeared to have a fibroblastic phenotype rather than an epithelial phenotype, and TDZ pretreatment prevent the TNF‐α/TGF‐β‐stimulated morphological alterations (Figure 6B). This outcome was evidenced by the clustering of the nuclei in the TNF‐α/TGF‐β administered group, as seen with a cells edges in with the Phalloidin images (Figure 8B). These findings confirm that TDZ can attenuate EMT, by inhibiting invasion and F‐actin distribution in TNF‐α/TGF‐β‐induced BEAS‐2B cells.

## 4 DISCUSSION

Epithelial‐mesenchymal transition is an crucial incident that increases the likelihood of cancer cell metastasis.^29^ Specifically, epithelial cells transition from extremely polarized, differentiated, cell‐cell into undifferentiated, isolated, mesenchyma‐like cells with invasive and migratory abilities. Additionally, during the progression of cancer, many tumour cells go through morphological and phenotypic changes that affect their plasticity. Numerous researchers have explored the actions of EMT in breast cancer.^12^, ^30^, ^31^ In breast cancer biopsies, the overexpression of mesenchymal markers associated with EMT is connected with augmented disease repetition, difficult clinicopathological outcomes, diminished survival and increased tumour fierceness. So, potential curative approaches must be recognized to minimize the breast cancer fierceness and avert the tumour growth. In the current exploration, we found that TDZ has anti‐metastatic and anti‐EMT potentials and distinguished the processes accountable for its influence on MDA‐MB‐231 cells. Also, we noticed E‐cadherin down‐regulation and changes in the EMT‐associated proteins, which were indicative of the instigation and maintenance of EMT mechanism in MDA‐MB‐231 cells. The anti‐EMT potential of TDZ pretreatment on breast cancer cells were suggested by its reestablishment of E‐cadherin protein expression.

The NF‐kB and PI3K/AKT pathways are concerned in frequent pathological progressions, including adhesion of cancer cells, angiogenesis, inflammation, and metastasis.^32^ The inhibition of NF‐κB stimulation represses uPA, VEGF, MMPs and tumour metastasis.^9^ To invade surrounding tissue, cancer cells must be able to transverse vessel walls, and to assist this event, they produce uPA, MMPs and VEGF, important components of cancer cell metastasis and invasion.^33^, ^34^, ^35^ In this exploration, we found that TDZ inhibits constitutively activated NF‐κB. The repression of NF‐κB function can block tumour initiation and metastasis and binding of related factors to these promoters. Suppression of emerges as appropriate for restraining the expression of uPA, MMP and VEGF. The data presented here reveal that the inhibition of MDA‐MB‐231 cell invasion with TDZ may occur partly through repression of uPA, MMP and VEGF expression via intonation of NF‐κB signalling pathways. Several preceding investigations were demonstrated that the PI3K/AKT signalling and their downstream factors are prime modulators of numerous aspects of cellular life including cell survival, multiplication, invasion and migration.^36^, ^37^ PI3K/AKT is targeted via the transcription factor NF‐κB and is linked with the invasion and metastasis of numerous cancers.^38^ In this investigation, we noticed that TDZ supplementation appreciably diminished the p65 protein expression that demonstrates TDZ suppresses NF‐κB function, possibly via decreasing stimulation of PI3K/AKT signalling in MDA‐MB‐231 cells. Accordingly, we consider that PI3K/AKT/NF‐κB inhibition contributes for the anti‐metastatic potentials of TDZ on breast cancer.

For the management of cancers, predominantly breast cancers, effective strategies include induction of apoptosis, chemical or biological agent‐mediated suppression of cell multiplication, and stimulation of cell cycle detain. Apoptosis‐stimulating agents are under exploration as a substitute agents for cancer management. One exploration revealed that supplementation of MDA‐MB‐231 cells along with carvacrol leads to the augmentation of the amount of cells in late apoptosis in a flow cytometry‐based examination of Annexin V/PI‐stained cells.^39^ In the current investigation, the supplementation of MDA‐MB‐231 cells along with TDZ appreciably diminished the cell viability. The characteristics of apoptosis, which includes caspase activation, chromatin condensation, internucleosomal DNA cleavage, and cellular morphology alterations.^40^ In this exploration, we noticed that supplementation of MDA‐MB‐231 cells with TDZ considerably augmented the cell viability linked to DNA fragmentation, suggesting an increase in apoptosis. In one study, treatment of cervical cancer cells with TDZ augmented the apoptotic cells.^21^ In a study of the effects of various TDZ analogues in A549 cells, the analogue GA‐13315 improved DNA fragmentation, caspase‐3 stimulation, and apoptosis.^41^ These results suggest that TDZ represses cancer cell growth and prompts the apoptosis.

Studies of several tumour cell replicas have shown that metastatic ability and tumour cell growth are dependent on MMP activity.^42^ Herein, we noticed that TDZ treatment can diminish the protein statuses such as uPA, uPAR and MMP‐9, in MDA‐MB‐231 cells that demonstrates that TDZ may be an anti‐metastatic agent in MDA‐MB‐231 cells. The VEGF expression is related to a destructive tumour actions and augmented angiogenesis.^34^ Altered epithelial cells are the main cause of VEGF discharge in the breast cancers. The inhibitory potential of TDZ on VEGF expression in MDA‐MB‐231 cells is suggestive of its negative regulation of neovascularization in breast tumours that would help to control tumour growth.

They comprise stem cells that renew to develop mammospheres during sequential passaging and progenitor cells, which undergo multi lineage variation. Accumulative data suggest that numerous cancers, for example breast cancer, are directed via a cellular sub‐population comprising cancer stem cells (CSCs), which regulates metastasis and resistant to the usual therapies. Accordingly, the prevention of CSC growth in breast cancer is an finest approach for preventing the metastasis and tumour development.^43^ Consequently, exploration on TDZ‐stimulated molecular events, which regulate CSC multiplication, is crucial to elucidate the anti‐metastatic and anticancer potentials of TDZ. Our exploration demonstrated that TDZ supplementation appreciably reduced mammosphere development and sphere. These outcomes show that TDZ inhibits mammosphere development.

Epithelial‐mesenchymal transition take place during the untimely phases of the conversion of tumours into the malignant neoplasm.^44^ The thought that EMT is allied with an progression of metastatic cancer cells is evidenced by the examination, which attainment of mesenchymal indicators, for example Snail, Vimentin and Twist by epithelial carcinoma cells is linked with augmented metastatic ability,^45^, ^46^, ^47^ as is increased nuclear expression of β‐catenin^9^ and E‐cadherin loss.^48^, ^49^ Furthermore, accumulative proof demonstrates that for many types of cancers, EMT can result in CSC transformation, drug resistance and a relatively poor prognosis. Therefore, pharmacological inhibition of EMT or inhibition of the functions of EMT transcription factors, for example Twist, ZEB1 and Snail, may be important to cancer management.^50^ In this examination, we found that TDZ treatment prevents EMT in metastatic BEAS‐2B cells. Our outcomes indicate the inhibition of EMT or inhibition of the functions of EMT transcription factors may be an additional process concerned in the antitumour potential of this compound.

In a hepatocellular carcinoma study, TGF‐β transforming activation has been shown to facilitate liver cancer lung metastases in mouse models.^28^ It remains to be known if the same mechanism is involved in lung metastases of breast cancer. In our examination, we also inspected the anti‐EMT and anti‐metastatic potentials of TDZ and the related processes in non‐tumorigenic BEAS‐2B cells stimulated with TNF‐α/TGF‐β. TGF‐β promotes tumour progression via stimulating EMT.^51^ TGF‐β‐stimulated EMT has the characteristics like attainment of a fibroblastic morphology, loss of E‐cadherin localization and augmented cellular motility.^52^ TNF‐α is a pro‐inflammatory mediator that performs a crucial roles in malignant tumour processes, for example invasion, motility and metastasis.^53^ TNF‐α induces EMT in renal cancer via repressing the E‐cadherin expression and enhancing Vimentin and MMP‐9 expressions. Activation with TGF‐β and TNF‐α may result in EMT‐like event, diminution of E‐cadherin expression in MDCK cells.^54^ In our study, we found that TDZ treatment of TNF‐α/TGF‐β‐triggered cells results in a up‐regulated E‐cadherin expression, and changes in EMT‐linked signalling regulators associated with the initiation and propagation of EMT. The advantage of pretreatment with TDZ lies in its ability to restore transcriptional and E‐cadherin enhancer action and prevent the decrease in epithelial markers stimulated via TNF‐α/TGF‐β stimulation. Moreover, we noted that the restoration of E‐cadherin promoter action is related to the NF‐κB, and MMP‐2/‐ 9 inhibitions, and inhibition of E‐cadherin promoter activity is a crucial molecular incident in the EMT inhibition stimulated via TNF‐α/TGF‐β. Our experimental results suggest that TDZ potentially restoration of E‐cadherin promoters.

The findings of the current study demonstrate the anti‐EMT and anti‐metastatic potentials of TDZ on human breast cancer (MDA‐MB‐231) cells. These abilities may due to the inhibition of NF‐κB signalling and modulation of PI3K/AKT and MMP9 signalling cascades, but additional investigation is still required to elucidate the relationships among NF‐κβ, PI3K/AKT, and MMP9 in response to TDZ treatment. These findings offer a novel outlook on the possible use of TDZ as an inhibitor of metastasis and EMT in breast cancer.

## CONFLICT OF INTEREST

No conflicting interest.

## AUTHOR CONTRIBUTIONS

**Peramaiyan Rajendran:** Conceptualization (lead); data curation (lead); funding acquisition (lead); investigation (lead); methodology (lead); project administration (lead); supervision (lead); writing‐original draft (lead). **Rebai Ben Ammar:** Conceptualization (supporting); investigation (supporting); writing‐review and editing (supporting). **Fatma J. Al‐Saeedi:** Investigation (supporting); validation (supporting); writing‐review and editing (supporting). **Maged Elsayed Mohamed:** Data curation (supporting); formal analysis (supporting); investigation (supporting); validation (supporting). **MIH Islam:** Data curation (supporting); formal analysis (supporting); validation (supporting); writing‐review and editing (supporting). **Saeed Y. Al‐Ramadan:** Resources (supporting); visualization (supporting); writing‐review and editing (supporting).

## Data Availability

The data that support the findings of this study are available from the corresponding author upon reasonable request.
